# Update on Extensively Drug-Resistant *Salmonella* Serotype Typhi Infections Among Travelers to or from Pakistan and Report of Ceftriaxone-Resistant *Salmonella* Serotype Typhi Infections Among Travelers to Iraq — United States, 2018–2019

**DOI:** 10.15585/mmwr.mm6920a2

**Published:** 2020-05-22

**Authors:** Louise K. François Watkins, Alison Winstead, Grace D. Appiah, Cindy R. Friedman, Felicita Medalla, Michael J. Hughes, Meseret G. Birhane, Zachary D. Schneider, Perrine Marcenac, Samir S. Hanna, Gauri Godbole, Kelly A . Walblay, Ashley E. Wiggington, Molly Leeper, Elizabeth H. Meservey, Kaitlin A. Tagg, Jessica C. Chen, Abdinasir Abubakar, Faris Lami, Asaad M. Asaad, Vickneswaran Sabaratnam, Aamer Ikram, Kristina M. Angelo, Allison Walker, Eric Mintz

**Affiliations:** ^1^Division of Foodborne, Waterborne, and Environmental Diseases, National Center for Emerging and Zoonotic Infectious Diseases, CDC; ^2^Epidemic Intelligence Service, CDC; ^3^Atlanta Research & Education Foundation, Atlanta, Georgia; ^4^Oak Ridge Institute for Science and Education, Oak Ridge, Tennessee; ^5^Tennessee Department of Health; ^6^Gastrointestinal Bacteria Reference Laboratory, Public Health England, London, United Kingdom; ^7^Illinois Department of Public Health; ^8^Missouri Department of Health and Senior Services; ^9^WDS, Inc., Suwanee, Georgia; ^10^World Health Organization Regional Office for the Eastern Mediterranean, Cairo, Egypt; ^11^Iraq Ministry of Health, Baghdad, Iraq; ^12^World Health Organization, Baghdad, Iraq; ^13^National Institute of Health, Islamabad, Pakistan; ^14^Division of Global Migration and Quarantine, National Center for Emerging and Zoonotic Infectious Diseases, CDC.

Ceftriaxone-resistant *Salmonella enterica* serotype Typhi (Typhi), the bacterium that causes typhoid fever, is a growing public health threat. Extensively drug-resistant (XDR) Typhi is resistant to ceftriaxone and other antibiotics used for treatment, including ampicillin, chloramphenicol, ciprofloxacin, and trimethoprim-sulfamethoxazole (*1*). In March 2018, CDC began enhanced surveillance for ceftriaxone-resistant Typhi in response to an ongoing outbreak of XDR typhoid fever in Pakistan. CDC had previously reported the first five cases of XDR Typhi in the United States among patients who had spent time in Pakistan (*2*). These illnesses represented the first cases of ceftriaxone-resistant Typhi documented in the United States (*3*). This report provides an update on U.S. cases of XDR typhoid fever linked to Pakistan and describes a new, unrelated cluster of ceftriaxone-resistant Typhi infections linked to Iraq. Travelers to areas with endemic Typhi should receive typhoid vaccination before traveling and adhere to safe food and water precautions (*4*). Treatment of patients with typhoid fever should be guided by antimicrobial susceptibility testing whenever possible (*5*), and clinicians should consider travel history when selecting empiric therapy.

Typhi is transmitted through the fecal-oral route, usually by contaminated water or food. The incubation period of typhoid fever is typically 6–30 days. Untreated, it has a mortality rate of 12%–30% (*3*,*4*). Ceftriaxone and ciprofloxacin are first-choice antibiotics, with ampicillin, azithromycin, or trimethoprim-sulfamethoxazole being alternative options (*5*). In the United States, typhoid fever is a notifiable disease, and approximately 350 culture-confirmed cases are submitted to CDC annually. Local and state public health departments send epidemiologic information from culture-confirmed cases to CDC’s National Typhoid and Paratyphoid Fever Surveillance system and submit isolates to CDC’s National Antimicrobial Resistance Monitoring System (NARMS) laboratory for antimicrobial susceptibility testing. Typhi isolates undergo whole genome sequencing (WGS) at public health laboratories when resources are available. WGS data are submitted to CDC’s PulseNet laboratory network and uploaded to the National Center for Biotechnology Information (NCBI).* WGS can be used to determine relatedness of isolates and to identify genes and mutations that confer resistance.

After XDR Typhi was reported in Pakistan, CDC initiated enhanced surveillance for ceftriaxone-resistant Typhi. CDC requested that health departments notify CDC immediately when a patient with typhoid fever reported recent travel to Pakistan. In addition, health departments used a supplementary interview form to collect additional information about travel and exposures for patients or household contacts who had been in Pakistan during the month before illness onset. The corresponding isolates underwent expedited antimicrobial susceptibility testing through NARMS. Finally, CDC implemented alert systems to automatically notify agency epidemiologists whenever a ceftriaxone-resistant Typhi isolate was identified by NARMS or reported to NCBI.

During January 1, 2016–August 31, 2019, CDC identified 96 Typhi infections among U.S. travelers to or from Pakistan (Figure 1). Among these, 30 (31%) isolates were identified as XDR by antimicrobial susceptibility testing (28) or WGS (two); isolates were resistant to ceftriaxone, ampicillin, chloramphenicol, ciprofloxacin, nalidixic acid, streptomycin, sulfisoxazole, and trimethoprim-sulfamethoxazole (Figure 2). The median age of patients with XDR typhoid fever was 11.5 years (range = 1–41 years), 53% (16 of 30) were male, 93% (26 of 28) were hospitalized, and none of 24 for whom information was available reported typhoid vaccination within 5 years of travel (Table). Among 20 patients with information on travel within Pakistan, 12 (60%) traveled to Karachi or other parts of Sindh province, the region of the reported XDR epidemic (*1*); the other eight (40%) patients did not report travel to Sindh but had visited Punjab province.

**FIGURE 1:**
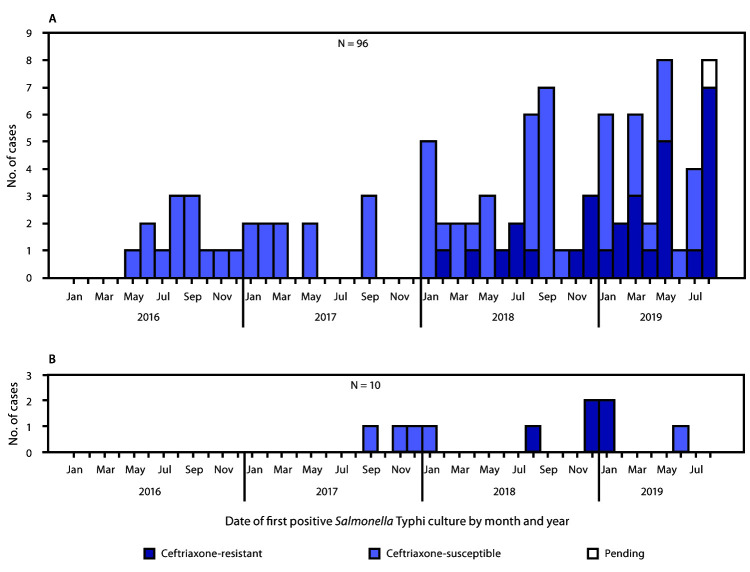
Cases of culture-confirmed typhoid fever linked to travel to or from Pakistan (A) or Iraq* (B) by ceftriaxone susceptibility status and culture date (month/year) — United States,^†^ January 1, 2016–August 31, 2019 * The patient whose case was diagnosed in November 2017 traveled to Iran only. ^†^ Two patients whose cases were diagnosed in January 2019 were residents of the United Kingdom who became ill after travel to Iraq (panel B).

**FIGURE 2:**
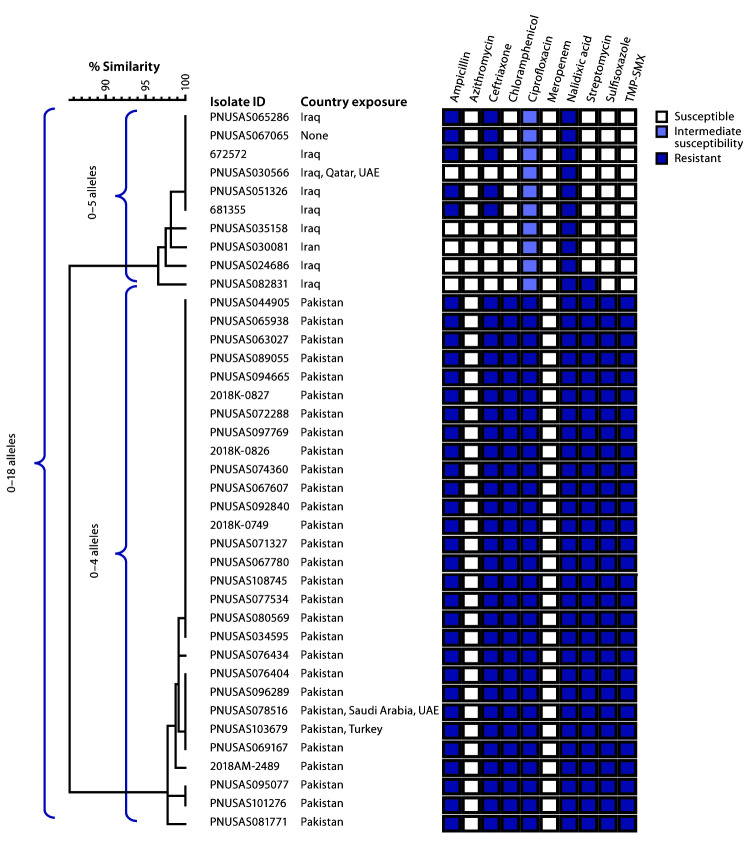
Core genome multilocus sequence typing (cgMLST) phylogenetic tree* of 39^†^ *Salmonella* Typhi isolates from two strains with ceftriaxone resistance among persons with travel to Iran, Iraq,^§^ and Pakistan — United States and United Kingdom,^¶^ 2017–2019. **Abbreviations:** TMP-SMX = trimethoprim-sulfamethoxazole; UAE = United Arab Emirates. * The tree was constructed using BioNumerics (version 7.6; Applied Maths). The National Center for Biotechnology Information strain identifier and country exposure(s) during the patient’s incubation period are shown. Shaded boxes indicate resistance patterns determined by antimicrobial susceptibility testing (n = 35) or predicted resistance from whole genome sequencing (n = 4, with isolate IDs 672572, 681355, PNUSAS095077, and PNUSAS101276). ^†^ One ceftriaxone-resistant Typhi isolate from a patient with travel to Pakistan was not included in this figure because the isolate did not undergo whole genome sequencing. ^§^ One isolate (ID PNUSAS067065) was cultured from a patient who did not travel, but whose asymptomatic father returned from Iraq in the 30 days before her symptom onset. ^¶^ Isolates with IDs 672572 and 681355 were cultured from residents of the United Kingdom.

**TABLE:** Characteristics of patients (n = 30) with extensively drug-resistant typhoid fever — United States 2018–2019

Characteristic (no. with available information)	No (%)
**Sex (30)**	
Male	16 (53)
**Age group (yrs) (30)**	
<2	1 (3)
2–5	6 (20)
6–11	8 (27)
12–17	5 (17)
18–41	10 (33)
**Purpose of travel (26)**	
Visiting friends and relatives	22 (85)
Other	4 (15)
**Destination within Pakistan (20)**	
Sindh province only	9 (45)
Sindh province and Punjab province	2 (10)
Sindh province, Punjab province, and Islamabad	1 (5)
Punjab province only	7 (35)
Punjab province and Islamabad	1 (5)
**Pretravel vaccination (24)**	0 (0)
**Hospitalization (28)**	26 (93)
Median duration of stay, days (range)	7.5 (2–19)
**Intensive care unit admission (19)**	3 (16)

In November 2018, CDC detected a ceftriaxone-resistant Typhi isolate with a novel resistance pattern in a patient who reported travel to Iraq in the 4 weeks preceding illness onset. As were the Pakistan XDR isolates, this isolate (PNUSAS051326) (Figure 2) was resistant to ceftriaxone, ampicillin, and nalidixic acid, but it showed intermediate susceptibility to ciprofloxacin and full susceptibility to other antibiotics, including chloramphenicol and trimethoprim-sulfamethoxazole.

Using information reported to NCBI, CDC identified nine additional isolates that were highly related to the isolate from the traveler to Iraq, corresponding to seven additional U.S. patients and two from the United Kingdom. Of these nine patients, seven (five U.S. residents and both U.K. patients) had also traveled to Iraq. One patient was a child who did not travel herself, but her father (who was asymptomatic) had returned from Iraq within the month before her illness began. One patient reported travel to Iran only. Of the 10 patients, nine were adults (median age = 43 years; range = 3–75 years), five were male, and none of six for whom information was available reported pretravel vaccination for typhoid. None reported travel to Pakistan. Specimen collection dates were from September 2017 through June 2019 (Figure 1); the five isolates clustered from August 2018 through January 2019 shared the same antibiotic resistance pattern, whereas the earlier and later isolates lacked resistance to ceftriaxone and ampicillin. No other U.S. cases of typhoid fever related to travel to Iran or Iraq have been reported to CDC since January 1, 2016.

Public health officials in Iraq noted an increase in cases of typhoid fever in the fall of 2018, which coincided with Arba’een, an annual religious pilgrimage to the city of Karbala, Iraq. Arba’een has been described as the world’s largest annual gathering (10–20 million participants). One of the British patients reported traveling to Iraq to attend Arba’een (*6*).

Genomic analysis showed that the strain of Typhi associated with travel to Iran and Iraq was genetically distinct from the XDR strain associated with travel to Pakistan (Figure 2). In both strains, ceftriaxone resistance was due to an extended-spectrum beta-lactamase resistance gene (*bla*_CTX-M-15_) carried by an IncY type plasmid. However, the plasmid found in the Pakistan strain (all travelers) and the plasmid found in the Iraq strain (five of 10 travelers) were not closely related.

As of August 31, 2019, all U.S. patients with ceftriaxone-resistant typhoid fever were linked to either Iraq or Pakistan. Ceftriaxone-resistant Typhi isolates in the United States during this period were susceptible to azithromycin and meropenem.

## Discussion

During February 2018–August 2019, CDC identified 33 ceftriaxone-resistant Typhi isolates from U.S. patients; no such isolates had been identified before 2018. Thirty isolates were from cases of XDR typhoid fever linked to travel to Pakistan; notably, these cases have occurred with increasing frequency and reflect the ongoing outbreak in Sindh province, with approximately 10,000 cases reported as of August 2019 (*7*). In November 2019, approximately 9.4 million children aged 9 months–15 years in Sindh province were vaccinated against typhoid fever with the typhoid conjugate vaccine prequalified by the World Health Organization (*8*). In August 2018, CDC identified a second strain of ceftriaxone-resistant Typhi, this one related to travel to Iraq. Isolates linked to travel to Iraq appear genetically distinct from isolates linked to travel to Pakistan, suggesting that the emergence of ceftriaxone resistance among these two strains was unrelated. In both strains, the ceftriaxone resistance is plasmid-mediated and has the potential to spread to other bacteria.

Eight U.S. patients with XDR Typhi linked to travel to Pakistan did not travel to Sindh province, suggesting the outbreak is more widespread in Pakistan than has been previously reported (*1*). Public health officials and clinicians should remain vigilant for cases of ceftriaxone-resistant Typhi in patients who have traveled to countries neighboring Iraq and Pakistan and of strains of Typhi with more extensive resistance, particularly to azithromycin, because this has been reported from some parts of South Asia (*9*). These cases also highlight the public health risks associated with mass gatherings such as the Arba’een pilgrimage. Public health authorities should prepare for mass gatherings by ensuring safe drinking water and food and adequate infrastructure for proper sanitation and hygiene (*10*).

The findings in this report are subject to at least three limitations. First, some cases of ceftriaxone-resistant Typhi that occurred before August 31, 2019, might have been missed because some health departments might have delayed both case reporting and isolate submission for susceptibility testing until the end of the 2019 calendar year. Second, detailed clinical or travel histories were not obtained for all patients because some patients did not respond to requests from the health department for more information. Finally, most clinical and travel information was obtained by patient self-report and not independently verified.

Currently, most Typhi infections diagnosed in the United States are not susceptible to fluoroquinolones, such as ciprofloxacin, and the prevalence of resistance is >10% for ampicillin, chloramphenicol, and trimethoprim-sulfamethoxazole (*3*). Therefore, ceftriaxone has become increasingly important for empiric treatment (*5*), and the emergence of ceftriaxone resistance in Typhi strains that are not susceptible to ciprofloxacin presents a significant treatment challenge. Clinicians should request antimicrobial susceptibility testing for all Typhi isolates and tailor patient treatment accordingly. All patients should be asked about travel, and special consideration should be given to empiric treatment for patients who have recently returned from Iraq or Pakistan. For XDR Typhi, azithromycin may be used for uncomplicated cases, and carbapenems (e.g., meropenem) may be used for severe illness (*2*). For travelers returning from Iraq, trimethoprim-sulfamethoxazole remains an alternative.

The emergence of ceftriaxone-resistant Typhi highlights the need for effective prevention measures. Notably, none of the patients for whom vaccine history was available had been vaccinated before travel. Clinicians should advise patients traveling to areas with endemic Typhi to receive pretravel typhoid vaccination and to practice safe food and water precautions (*4*). Additional information is available at https://www.cdc.gov/typhoid-fever/prevention.html (prevention measures for travelers), https://www.cdc.gov/typhoid-fever/resources.html, (resources for the public, public health officials, and clinicians) and https://wwwnc.cdc.gov/travel/diseases/typhoid (disease and travel-specific information).

**Summary**

**What is already known about this topic?**

Before 2018, no ceftriaxone-resistant *Salmonella* Typhi cases had been identified in the United States. Extensively drug-resistant *Salmonella* Typhi, susceptible only to azithromycin and carbapenems, has caused a typhoid fever outbreak in Pakistan since 2016.

**What is added by this report?**

During February 2018–August 2019, 33 cases of ceftriaxone-resistant *Salmonella* Typhi were detected in the United States. Whole genome sequencing of isolates identified two distinct clusters, associated with travel to Pakistan (30 cases) and Iraq (three).

**What are the implications for public health practice?**

Vaccination and food and water precautions can help prevent typhoid fever. Clinicians and public health officials should remain vigilant for ceftriaxone-resistant Typhi in patients who have traveled to Pakistan, Iraq, or neighboring countries.
